# A protocol of a randomized controlled multicenter trial for surgical treatment of lumbar spondylolisthesis: the Lumbar Interbody Fusion Trial (LIFT)

**DOI:** 10.1186/s12891-016-1280-8

**Published:** 2016-10-06

**Authors:** Suzanne L. de Kunder, Kim Rijkers, Sander M. J. van Kuijk, Silvia M. A. A. Evers, Rob A. de Bie, Henk van Santbrink

**Affiliations:** 1 Department of Neurosurgery, Maastricht University Medical Center, Postbus 5800, 6202 AZ Maastricht, The Netherlands; 2 Department of Neurosurgery, Zuyderland Medical Center, Heerlen, The Netherlands; 3 Department of Clinical Epidemiology and Medical Technology Assessment (KEMTA), Maastricht University Medical Center, Maastricht, The Netherlands; 4 Department of Health Services Research, Maastricht University, Maastricht, The Netherlands; 5 CAPHRI School for Public Health and Primary Care, Maastricht University, Maastricht, The Netherlands; 6 Department of Epidemiology, Maastricht University, Maastricht, The Netherlands

## Abstract

**Background:**

With a steep increase in the number of instrumented spinal fusion procedures, there is a need for comparative data to develop evidence based treatment recommendations. Currently, the available data on cost and clinical effectiveness of the two most frequently performed surgeries for lumbar spondylolisthesis, transforaminal lumbar interbody fusion (TLIF) and posterior lumbar interbody fusion (PLIF), are not sufficient. Therefore, current guidelines do not advise which is the most appropriate surgical treatment strategy for these patients. Non-randomized studies comparing TLIF and PLIF moreover suggest that TLIF is associated with fewer complications, less blood loss, shorter surgical time and hospital duration. TLIF may therefore be more cost-effective. The results of this study will provide knowledge on short- and long-term clinical and economical effects of TLIF and PLIF procedures, which will lead to recommendations for treating patients with lumbar spondylolisthesis.

**Methods:**

Multicenter blinded Randomized Controlled Trial (RCT; blinding for the patient and statistician, not for the clinician and researcher). A total of 144 patients over 18 years old with symptomatic single level lumbar degenerative, isthmic or iatrogenic spondylolisthesis whom are candidates for LIF (lumbar interbody fusion) surgery through a posterior approach will be randomly allocated to TLIF or PLIF. The study will consist of three parts: 1) a clinical effectiveness study, 2) a cost-effectiveness study, and 3) a process evaluation.

The primary clinical outcome measures are: change in disability measured with Oswestry Disability Index (ODI) and change in quality adjusted life years (QALY) measured with EQ-5D-5L. Secondary clinical outcome measures are: Short Form (36) Health Survey (SF-36), VAS back pain, VAS leg pain, Hospital Anxiety Depression Scale (HADS), complications, productivity related costs (iPCQ) and medical costs (iMCQ). Measurements will be carried out at five fixed time points (pre-operatively and at 3, 6, 12 and 24 months).

**Discussion:**

It is hypothesized that TLIF, compared to PLIF, has similar clinical outcome or is possibly better in reducing disability. Moreover, direct medical costs are expected to be lower due to less surgical morbidity, shorter hospital stay and shorter surgical time. Indirect costs are assumed to be lower for TLIF as well, because we suspect less working days are lost. Currently, prospective data comparing clinical and cost-effectiveness of both techniques are not available. Therefore, in clinical practice both techniques are used and the choice for technique is greatly based on surgeon’s preference. The demand for spinal fusion surgery has risen steeply over the last 10 years and is expected to increase even further in the near future. As a result, the burden on society (and the working population) will increase. In case our hypothesis is confirmed, treatment guidelines will be adapted, and TLIF will be recommended as first choice surgical treatment of lumbar spondylolisthesis. Ultimately this will lead to reduction of (direct and indirect) costs and better clinical outcome for spondylolisthesis patients eligible for instrumented spinal surgery.

**Trial registration number:**

Netherlands Trial Registry, number 5722 (registration date March 30, 2016).

## Background

Neurogenic leg pain is a frequent complaint in the general population. This pain is can be caused by compression or stretch of nerve roots or cauda equina fibers (lumbar radiculopathy or neurogenic claudication respectively). Lumbar disc herniation and spinal canal stenosis are the classic and most common causes. An other cause of neurogenic leg pain is becoming more and more prevalent, namely lumbar spondylolisthesis [1]. If conservative treatment for neurogenic leg pain fails, surgical treatment can be considered. In case of lumbar disc herniation or spinal canal stenosis, decompression surgery is executed. In case of spondylolisthesis, decompression alone is not sufficient, and additional spinal fusion is recommended and common practice nowadays. In the US, between 1998 and 2008 the national bill for instrumented spinal fusion has increased 7.9-fold [2, 3]. This only will increase further in the next decades with an aging population.

A number of surgical techniques for spinal fusion are available. Of these, transforaminal lumbar interbody fusion (TLIF) and posterior lumbar interbody fusion (PLIF) are most frequently performed in the Netherlands. Both procedures consist of pedicle screw placement. In the TLIF procedure, this is followed by placement of one cage in the intervertebral space using a unilateral approach. The PLIF procedure consists of placement two identical cages bilaterally in the intervertebral space using a bilateral approach.

There are no strict indications for using either techniques, because a number of prospective studies have shown that both methods effectively reduce leg pain [3–7]. As a result, the choice of technique is greatly based on surgeon’s preference. Even though these techniques are assumed to be equal, nonrandomized studies and one small RCT comparing TLIF and PLIF suggest that TLIF is associated with fewer complications, less blood loss, shorter surgical time and hospital duration [8–10]. Our own retrospective data of 254 TLIF and PLIF patients confirm this, and additionally reveal that TLIF patients score better on different quality of life related outcome parameters (SF-36, ODI) compared to PLIF [11]. These findings have not been confirmed in a randomized controlled trial.

However, with a steep increase in the number of instrumented spinal fusion procedures there is a need for comparative data to develop evidence based treatment recommendations.

This study proposes to analyse in a high quality design (multicenter prospective randomized controlled trial) effectiveness and cost-effectiveness of the TLIF technique compared to PLIF technique for patients with leg pain caused by single level lumbar spondylolisthesis.

## Methods

This study consists of three parts, each with its own research question:

I. Clinical effectiveness
  1) Is transforaminal lumbar interbody fusion (TLIF) effective in reducing disability in comparison to posterior lumbar interbody fusion (PLIF) in patients with single level lumbar spondylolisthesis?
II. Cost-effectiveness
  2) Is transforaminal lumbar interbody fusion (TLIF) cost-effective in comparison to posterior lumbar interbody fusion (PLIF) in patients with single level lumbar spondylolisthesis from a societal perspective?
III. Process evaluation
  3) What are the experiences and opinions of patients and professionals regarding TLIF?

### Design

A nation wide, prospective, multicenter, patient blinded, randomized controlled superiority trial. Patients will be randomized into one of two parallel groups (1) TLIF and (2) PLIF in a 1:1 ratio. The study inclusion period will be approximately 2 years, and the follow-up period 2 years (total study duration 4 years). Informed consent will be acquired from all participants. The study has been approved by the local institutional medical ethical committee (Medical Research Ethics Committee Zuyderland, METC 16-T-36) and has been registered with the Netherlands Trial Registry, part of the Dutch Cochrane Centre (number 5722).

### Study population

One hundred forty four eligible lumbar spondylolisthesis patients will be included in this study. Eligible are patients with:

- Indication for LIF (lumbar interbody fusion) surgery through a posterior approach
- Clinical mono uni- or bilateral lumbar radiculopathy or intermittent neurogenic claudication caused by a single level isthmic, degenerative or iatrogenic spondylolisthesis grade I, II or III at level L3L4, L4L5 or L5S1.
- Single level spondylolisthesis with central or foraminal stenosis on MRI (or CT), of which the anatomical level is corresponding the clinical syndrome.
- Age over 18 years.
- Psychosocially, mentally, and physically able to fully comply with this study protocol.
- Written informed consent prior to this study.

Patients will be excluded of participation in this study when any of the following criteria are met:

- Previous radiotherapy at the intended surgical level.
- (Progressive) motor failure and/or anal sphincter disorders which urges instant intervention.
- Active infection.
- Immature bone (ongoing growth).
- Active malignancy.
- Pregnancy.
- Symptomatic osteoporosis.
- Contra-indications for anesthesia or surgery.
- Inadequate command of the Dutch language.

### Setting and recruitment

This is a cooperating project involving six Dutch hospitals (Maastricht University Medical Center - Maastricht, Zuyderland Medical Center - Heerlen, University Medical Center Groningen - Groningen, Radboud university medical center - Nijmegen, Canisius Wilhelmina Hospital - Nijmegen and Isala - Zwolle). These hospitals have been chosen because of their high volume of instrumented spine surgery and their familiarity with TLIF and PLIF. Patients referred to the outpatient clinic with an indication for LIF surgery are eligible to participate in the study, and will be referred by colleagues to the researchers.

Researchers will inform the patient verbally and in writing. When the patient is willing to participate (patients are allowed to use a cooling off period of one week) an informed consent form will be signed by the patient and the researcher, and patients will be allocated randomly to either the TLIF or PLIF group.

### Sample size calculation

The difference in ODI improvement is defined as primary endpoint and will be used for calculating sample size. An improvement of seven points is considered a minimal clinically important difference [12]. Based on own retrospective data, ODI improvement after TLIF was 17.5 points (35 %), and 9.5 points (19 %) after PLIF. The response within each subject group was normally distributed with standard deviation of 16. Assuming that a true difference between the experimental and control group-means is at least eight, we will need to study 64 experimental subjects and 64 control subjects to be able to reject the null hypothesis that the population means of the experimental and control groups are equal with probability (power) of 0,8. The Type I error probability associated with this test of this null hypothesis is 0,05. Based on a 10 % loss to follow-up, we intend to include 144 patients (72 patients per group).

### Randomization

Participants will be randomly assigned by the researcher to either the TLIF or PLIF group with an 1:1 allocation using a web based computer generated randomization schedule stratified by treatment hospital and type of spondylolisthesis by variable block algorithm with random blocks of four, six or eight.

Patients are kept blinded for the allocated treatment during the follow-up period of two years.

At the end of the follow-up the blind can be lifted upon the patient’s request. The statistician is blinded as well.

### Interventions

#### TLIF group

The patient undergoes standard surgical treatment of degenerative listhesis with central spinal canal stenosis, or of isthmic listhesis with foramen stenosis. All patients receive antibiotic prophylaxis according to local hospital protocol. After receiving antibiotic prophylaxis, the patient is brought under general anesthesia and positioned prone. A midline or paramedian posterior approach is performed, exposing the posterior lumbar elements including the facet joints. Poly-axial pedicle screws are placed bilaterally, using fluoroscopic guidance or navigation, depending on preference of the surgeon. In case of spinal canal stenosis, the central part of the spinal canal is decompressed by laminectomy. Unilateral exposure to the intervertebral disc is assured by total unilateral facetectomy, decompressing the descending and leaving roots. In the case of bilateral symptomatic leg pain, the side of the unilateral approach is free of choice for the surgeon. Unilateral facetectomy is performed to gain access to the intervertebral disc. Discectomy is performed. Endplate cartilage is prepared to provide a host bed of bleeding subchrondral bone for placement of the cage. The TLIF cage size is determined by a trial cage and fluoroscopy. The definitive cage is filled with autologous bone or allograft and is tamped into place. Its position is checked radiologically. After placement of the TLIF cage, the remainder of the disc space is filled with autologous bone, obtained from the decompression. A titanium rod interconnects the screws on each side. The spreader is removed and the wound is thoroughly irrigated and closed in several layers without suction drainage.

#### PLIF group

Pedicle screw placement and if necessary, laminectomy as in the TLIF group. Bilateral access to the intervertebral disc assured by resection of the pars articularis inferior and partial resection of the pars superior of the facet joint. Bilateral discectomy is performed. Subsequently, endplate cartilage is prepared to provide a host bed of bleeding subchrondral bone for placement of the cages. Determination of cage size by trail cages and fluoroscopy. Before placement of the definitive cages, the disc space is partially filled with autologous bone, obtained from decompression. The definitive cages are also filled with autologous bone or allograft and are tamped into place with fluoroscopic guidance. Their position is checked radiologically. A titanium rod interconnects the screws bilaterally. The wound is closed in the same matter as in the TLIF group.

**Fig. 1:**
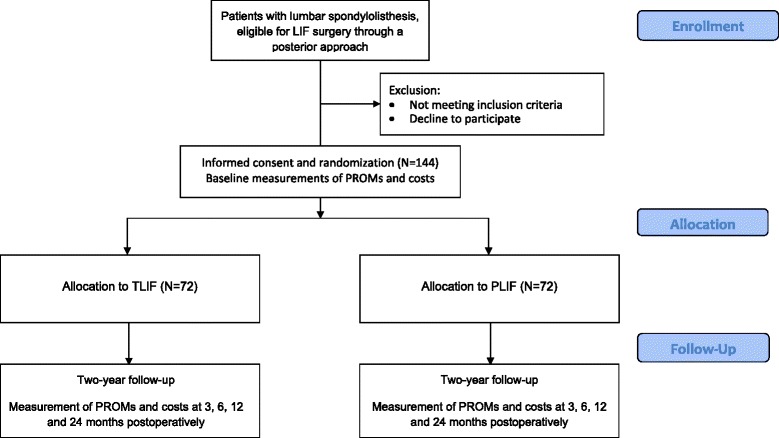
Study design flow chart

The flow of patients through the study is summarized in Fig. 1.

### Post-surgical care

Standardly there is no postoperative administration of IV antibiotics. Position of the implants will be checked by means of lumbar spine X-ray (anterior-posterior and lateral). Patients are encouraged to mobilise, initially with guidance of a physiotherapist, and resume daily activities as soon as possible. No additional physical therapy at home is advised.

### (Clinical) effectiveness

To assess the (clinical) effectiveness of both procedures, patients are asked to fill out web based questionnaires concerning Patient Related Outcome Measurements (PROMS) at five fixed time-points, namely preoperatively and 3, 6, 12 and 24 months postoperatively.

#### Primary outcomes

Change in disability measured using the Oswestry Disability Index (ODI) [13] and change in quality adjusted life years (QALY) assed with EQ-5D-5L [14].

#### Secondary outcomes

Quality of life will be further assed using the Short Form (36) Health Survey (SF-36) [15].

Pain will be measured using the Visual Analogue Scale (VAS) score for back pain and leg pain [16].

The degree/presence of preoperative anxiety and depression will be measured using the Hospital Anxiety Depression Scale (HADS) [17]. Societal costs will be measured retrospectively with the a Medical Cost Questionnaire (iMCQ) and the Productivity related Cost Questionnaire (iPCQ) [18].

Direct and indirect surgical complications including dural tear, postoperative infection, deep venous thrombosis, hematoma, hardware failure, neurological deficits, medical other complications as pneumonia or urinary tract infection will be registered.

#### Other study parameters

Sex, age, BMI, smoking habits, occurrence of diabetes, diagnosis, level, grade of spondylolisthesis, previous back surgery and ASA classification. Perioperative morbidity will be correlated to use of antibiotics, duration of surgery, intraoperative blood loss and duration of hospitalization.

#### Economic evaluation

The economic evaluation will assess cost-effectiveness (CEA) and cost-utility (CUA) from a societal and health care perspective. Costs will be related to change in disability measured with ODI and change in cost-effectiveness analysis and to changes in quality-adjusted life years (QALYs) with EQ-5D-5L in the cost-utility analysis. The analysis will be performed with a time horizon of two years.

Data are collected in web based CRFs (case report forms) and by means of questionnaires. Included costs consist of: 1. health care costs, 2. patient and family costs and 3. other costs. Healthcare costs are for example costs of surgical intervention (either TLIF or PLIF), hospital care (including costs for treating complications), medication, outpatient visits and resource use outside the hospital such as general practitioner visits and physical therapist visits. Among patient and family costs are travel costs, informal care and home care. Other costs are costs such as productivity losses due to absence from work. Information on these costs will be collected with a questionnaire designed for consumption of healthcare in the Dutch system (Medical Consumption Questionnaire - iMCQ) and a questionnaire designed for productivity costs in the Dutch system (Productivity Costs Questionnaire iPCQ). Both questionnaires have a recall period of 3 months and will be administered repeatedly at five fixed time points.

#### Process evaluation

To assess the experiences and opinions of patients and professionals with TLIF a process evaluation according to the framework provided by Saunders will be performed [19].

This framework consists of a stepwise approach in which important characteristics for the process-evaluation plan are identified along seven basic components, namely: fidelity (quality), dose delivered (completeness), dose received (exposure), dose received (satisfaction), reach (participation rate), recruitment and context.

For this process evaluation both qualitative and quantitative data will be collected. At the end of the study a short interview will be held with the principal investigator of every participating center, where the investigator can reflect on his/her experiences with the surgical techniques. A patient board is set up to ensure patient representation. Patients will be questioned using a semi-structured questionnaire covering the topics identified in the framework provided by Saunders et al. [19].

### Analysis

#### Clinical effectiveness

Data will be analysed according to the intention-to-treat principle. Difference in ODI change and EQ-5D-5L between baseline and subsequent measurements will be analysed using analysis of covariance (ANCOVA) to correct the effect of intervention as compared to controls for potential baseline differences and to gain precision in the effect estimates. In addition, we will use linear mixed models to analyse changes within the treatment groups as well as differences between the intervention and control group in the ODI and EQ-5D-5L over time.

Linear mixed models will also be used to analyse changes on secondary outcome measurements over time both within and between groups (Short Form (36) Health Survey, VAS back pain and leg pain and Hospital Anxiety Depression Scale (HADS)). Multivariable linear regression analysis will be performed to determine differences in change scores between the two groups at fixed time points.

Differences in the proportion of participants that report complications over the study period (up to 24 months), will be evaluated by means of logistic regression analysis.

All results will be presented as absolute mean differences with 95 % confidence intervals, or odds ratios with 95 % confidence intervals.

#### Economical evaluation

Costs will be linearly interpolated to estimate total costs covered by the time period between consecutive assessments. Unit prices will be determined according to Dutch guidelines, expressed in 2016 Euros and will be indexed if necessary using consumer price indices. Otherwise, integral cost-prices will be obtained from the Maastricht University Medical Center, or cost-price calculations will be performed.

#### Patient outcome analysis

The primary clinical outcome is the change in disability measured with ODI, over the course of a two year follow-up period, to which total societal costs will be related in the CEA. For the CUA, utilities are assessed using the EQ-5D-5L [14]. These utilities will be converted following the area under the curve method into QALYs using the United Kingdom social tariffs. Changes in QALYs over the course of the two-year follow-up period will be related to total societal costs in the CUA [20].

Costs and effects will be discounted according to Dutch pharmaco-economic guidelines. Standard sensitivity analyses and bootstrap analysis will be performed to investigate the uncertainty surrounding the cost-effectiveness ratios [21]. Based on the bootstrap results, cost-effectiveness acceptability curves will be constructed, showing threshold values for a wide range of cost-effectiveness, the probability that TLIF is more cost-effective.

In addition to the CEA and CUA, a model-based simulation approach will be used to assess generalizability of the findings.

The Budget Impact Analyses (BIA), alongside the CEA, will be performed to address the financial consequences of implementing the most cost-effective treatment as intervention of choice in patients with lumbar spondylolisthesis. The BIA is based on the results of the clinical trial and will be conducted according to the ISPOR guidelines and Dutch guideline for executing economic evaluations in health care [20, 22] from various perspectives: (i) wider societal perspective, i.e. including productivity losses; (ii) a narrower perspective of the public purse (in Dutch: Budgettair Kader Zorg (BKZ)); (iii) the perspective of the health care insurer. All scenarios will be compared with a reference scenario which consists of the current standard of performing both TLIF and PLIF. The BIA will be estimated for various implementation levels (10, 25, 50 and 100 % of the intended target group). Furthermore, scenarios will be modelled in which the timeline of implementing the most cost-effective treatment as intervention of choice in 100 % of the hospitals is varied between direct implementation to implementation in five years.

#### Process evaluation

Quantitative data will be analysed with appropriate statistical testing; descriptive statistics, Chi square tests and ANOVA. Data from focus groups and interviews will be categorized, so relevant themes can be identified.

## Discussion

This study will determine the clinical effectiveness and cost-effectiveness of TLIF compared to PLIF for patients with leg pain caused by single level lumbar spondylolisthesis. The demand for spinal fusion surgery has risen steeply over the last ten years and is expected to increase even further in the near future. In times of rising health care costs and resulting budget limitations, there is a need for solid, comparative, cost-effectiveness studies to be able to recommend the best choice, clinically as well as cost-effectively, of surgery for these patients. It is hypothesized that TLIF, compared to PLIF, is superior in reducing disability and thus has a better clinical outcome. Moreover, health care costs are suspected to be lower due to less surgical morbidity, shorter hospital stay and shorter surgical time. Productivity losses are assumed to be lower for TLIF as well, because less working days are lost.

Currently, the choice for technique is greatly based on surgeon’s experience and preference. The strength of this multicenter study is that because of randomization, the preference of the surgeon no longer determines which technique is used. Also this study is, to our knowledge, the first where cost-effectiveness of both procedures will be explored and compared.

One of the limitations of this study is the sample size. We do expect to be able to draw conclusions on the primary outcomes. However, for some of the secondary outcomes (for example complications) the sample size will be too small. We aim to see if results are comparable with those previously reported in literature.

Additionally, we will perform a process evaluation to assess the experiences and opinions of patients and professionals with TLIF.

In case our hypothesis is confirmed, this could lead to reduction of (healthcare and productivity losses) costs and better clinical outcome for spondylolisthesis patients eligible for instrumented spinal surgery. Recommendations considering the best choice will be very helpful for spine surgeons in the future and lead to adaptation of the current Dutch guidelines.

## Abbreviations

ANCOVA: Analysis of covariance
ASA: The American Society of Anesthesiologists
BIA: Budget impact analysis
BKZ: Budgettair Kader Zorg
CEA: Cost-effectiveness analysis
CRF: Case report form
CT: Computed tomography
CUA: Cost-utility analysis
EQ-5D-5L: EuroQol five dimensions questionnaire with five-level scale
HADS: Hospital anxiety depression scale
IC: Informed consent
iMCQ: Institute for Medical Technology Assessment - Medical Consumption Questionnaire
iPCQ: Institute for Medical Technology Assessment - Productivity Cost Questionnaire
LIF: Lumbar interbody fusion
MREC: Medical research ethics committee
MRI: Magnetic resonance imaging
ODI: Oswestry disability index
PLIF: Posterior lumbar interbody fusion
PROMs: Patient related outcome measurements
QALY: Quality adjusted life year
RCT: Randomized controlled trial
SF-36: Short Form (36) Health Survey
TLIF: Transforaminal lumbar interbody fusion
VAS: Visual analog scale
